# Single-Lung Transplantation in the Setting of Aborted Bilateral Lung Transplantation

**DOI:** 10.1155/2011/535649

**Published:** 2011-06-07

**Authors:** Varun Puri, Tracey Guthrie, Masina Scavuzzo, Daniel Kreisel, Alexander S. Krupnick, G. Alexander Patterson, Bryan F. Meyers

**Affiliations:** ^1^Department of Surgery, Washington University School of Medicine, St. Louis, MO 63110, USA; ^2^Division of Cardiothoracic Surgery, Barnes Jewish Hospital, One Barnes Jewish Hospital Plaza, 3108 Queeny Tower, St. Louis, MO 63110, USA

## Abstract

*Background*. The outcome of patients undergoing a single-lung transplant in the setting of an aborted bilateral lung transplant is unclear. *Methods*. A retrospective review of single lung transplants at an institutional program. *Results*. Of the 543 lung transplants performed over the last 10 years, 31 (5.7%) were single-lung transplants. Nineteen of 31 (61%) were planned single-lung transplants, while 12/31 (39%) were intraoperatively aborted, double lung transplants converted to single-lung transplants. The aborted and planned groups were similar in age, lung allocation score and NYHA status. The reasons for aborted double lung transplantation were cardiac/hemodynamic instability 4/12 (33%), difficult pneumonectomy 3/12 (25%), size mismatch 4/12(33%), and technical issues 1/12 (8%). The aborted group had higher CPB utilization (5/12 versus 1/19, *P* = .02), similar ischemic times (260 versus 234 min) and similar incidence of grade 3 primary graft dysfunction (6/12 versus 3/19, *P* = .13). ECMO was required for graft dysfunction in 2 patients in the aborted group. The one and two-year survival was 84% and 79% in the planned group and 62% and 52% in the aborted group, respectively. *Conclusions*. Patients undergoing single-lung transplantation in the setting of an aborted bilateral lung transplant may be at a higher risk of worse outcomes.

## 1. Background

More than two thousand five hundred lung transplants were performed worldwide in 2007, and the number has been gradually increasing over the last decade [1]. Two common diagnoses for lung transplant recipients, emphysema and pulmonary fibrosis, may be treated by either single, or bilateral lung transplantation. Proponents of either approach have good arguments in their favor. In general, patients undergoing single-lung transplantation (SLT) have similar short-term and slightly lower long-term survival compared to bilateral lung transplantation (BLT) [1, 2]. 

Usually, the decision to implant one lung or to perform a bilateral transplant is made prior to the operation. Individual center preference, patient factors, and whether one or two donor lungs are being offered are important variables in the decision making process. Our anecdotal experience has shown that some patients ultimately undergo SLT in the setting of an aborted BLT. Registry data and published literature do not separately report this population and their outcomes are clubbed with the SLT cohort. This led us to investigate our institutional database for outcomes in patients undergoing SLT in the setting of aborted BLT.

## 2. Patients and Methods

We performed a retrospective review of a prospectively maintained institutional adult lung transplantation database from January 2000 through December 2009. All patients who had undergone SLT were reviewed. Demographic data, perioperative data and outcomes were abstracted. All operative reports were reviewed to establish if the operation was a planned SLT or an aborted BLT. Specific causes for the intraoperative decision were recorded. Operative mortality included those patients who died within the first 30 days after the operation or later during the same hospitalization.

Comparisons between the two groups were made with unpaired, two-tailed *t* tests for means of normally distributed continuous variables and Wilcoxon rank sum tests for skewed data. Either *X* ^2^ or Fisher exact tests were used to compare categorical data. Kaplan-Meier analysis was used to estimate survival. Followup was censored for end of study and expiration. All data analyses were done using SPSS (SPSS 13.0 for Windows; SPSS Inc, Chicago, Ill). *P*  value less than  .05 was considered statistically significant, and adjustments were not made for multiple comparisons.

## 3. Results

Of the 543 lung transplants performed from 01/2000 to 12/2009, thirty-one (5.7%) were single-lung transplants. Nineteen of 31 (61%) were planned single-lung transplants while 12/31 (39%) were intraoperatively aborted, double lung transplants converted to single-lung transplants. The aborted and planned groups were similar in age, gender, mean lung allocation score, waiting list time, preoperative NYHA status, and mean pulmonary artery pressure (data not shown). All donors were evaluated with the standard criteria [3], and extended criteria donors were not utilized. The donors for the two groups were comparable in age and PaO_2_ (data not shown). Forty two percent of donors in the aborted group were distant compared to 11% in the planned group (*P* = .078), and this did not result in a difference in cold ischemia times. (Table 1)

The diagnoses in the aborted group were emphysema 5/12 (42%), Pulmonary fibrosis 5/12 (42%), others 2/12 (16%) (Bronchiolitis obliterans syndrome 1, Lymphangioleiomyomatosis 1) and were similar to the planned group. 

**Figure 1:**
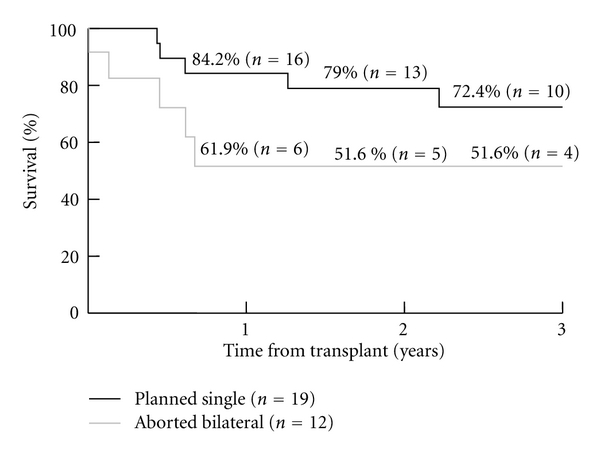
Kaplan Meier Survival stratified by Groups.

The reasons for aborted double lung transplantation were cardiac/hemodynamic instability 4/12 (33%), difficult pneumonectomy 3/12 (25%), size mismatch 4/12 (33%), and technical issues with atrial anastomosis 1/12 (8%). In comparison with the planned group, the aborted group had higher CPB utilization and higher incidence of secondary chest closure (Table 1). Two patients in the aborted group required ECMO support for severe PGD (primary graft dysfunction). The only perioperative death occurred in the aborted group. The groups were similar in early postoperative outcomes of PGD, length of mechanical ventilation and airway complications (Table 2). The median hospital stay was 23 days for the aborted group versus 13 days for the planned group (*P* = .067). The one- and two-year survival was 84% and 79% in the planned group and 62% and 52% in the aborted group, respectively (Table 2, Figure 1).

In all our cases in the aborted group, the issues precipitating the decision were technical (pleural adhesions, bleeding) or physiologic (hemodynamic instability, poor oxygenation). In many instances, more than one factor was considered. In general, two patterns were recognized. The first group of patients (*n* = 7) had fused pleural spaces or an overwhelming mismatch in function between the two native lungs, and an oversized donor lung that had good function immediately after implantation in place of the native lung with poorer function. The decision to implant only one lung was made early in most of these patients, and they generally had a smooth postoperative course. The second group of patients (*n* = 5) presented with significant bleeding or hemodynamic problems during the first pneumonectomy or implantation, usually requiring urgent cardiopulmonary bypass. These patients generally had a difficult early postoperative course often with significant transfusion requirements, reoperation for bleeding, early respiratory failure, and the need for ECMO in two cases. Although no specific preoperative risk factors predicted the need to abort a BLT and perform a SLT in our patients, a review of the operative records indicated that an early decision to implant only one lung led to smoother postoperative course

## 4. Discussion

The decision to perform BLT or SLT is often dependent upon institutional preference. The proponents of SLT argue that it is an easier procedure to perform, has less morbidity and mortality associated with it, and results in shorter ischemic time. There is also the societal benefit of two recipients benefiting from one donor. Proponents of BLT have argued that BLTs result in fewer ventilation/perfusion mismatches, are easier to care for in the perioperative period, will provide better overall lung function, are protective against the physiologic manifestations of obliterative bronchiolitis, and offer a better long-term survival [1, 2, 4–6]. Additionally, the longer ischemic times associated with BLT have not led to clinical sequelae [7, 8]. In patients with pulmonary fibrosis, SLT seems to have short-term survival benefit but long-term harm; whereas BLT seems to have short-term harm but long-term survival benefit [9]. BLT leads to longer survival than SLT in patients with COPD, especially those who are younger than 60 years [10].

Recent registry data have shown that the utilization of BLT is on the rise [1, 2]. Unadjusted registry data also show that BLT may provide intermediate term survival advantage over SLT double lung conditional 1/2-life = 9.0 years versus single-lung conditional 1/2-life = 6.4 Years where conditional half life is the time to 50% survival for the subset of recipients who were alive at 1 year after transplantation. We preferentially perform BLT whenever feasible. However, owing to the limited number of organs available, we recognize that SLT is a reasonable option for older patients with COPD and IPF [11]. It is unlikely that the debate between SLT and BLT proponents will be conclusively settled in the near future [12, 13]. In this setting, ongoing analyses of outcomes for both groups and subgroups would be useful.

Thirty-nine percent of the SLTs we performed over the study period were in the setting of aborted BLTs. This number is likely to be lower at institutions where SLT is performed more frequently or preferentially for emphysema and pulmonary fibrosis. Published literature and registry data do not have any information on the true incidence of this scenario. We were able to show a higher need for cardiopulmonary bypass, delayed chest closure, and a trend towards longer ICU and hospital stay in the aborted group. Also, despite the survival curves appearing divergent, statistical difference was not achieved between the two groups. This is likely related to the small number of patients available for analysis.

Outcomes in lung transplantation are predicated upon technical success. We are now prospectively recording episodes of intraoperative conversion from BLT to SLT in our institutional database. Collection of similar data at registry level will provide an insight into the incidence of this event and clinical scenarios where it occurs most frequently. This will be useful in not only better planning for a smoother operation at institutional level but may lead to a societal benefit with two planned single-lung transplants occurring instead of one single-lung transplant in an aborted planned bilateral procedure.

## 5. Conclusions

Patients undergoing single-lung transplantation in the setting of an aborted bilateral lung transplant may be at a higher risk of worse outcomes. In outcomes analyses, this subset of single-lung transplants should be identified.

## Figures and Tables

**Table 1:** Operative data.

Variable	Aborted bilateral (*n* = 12)	Planned single (*n* = 19)	*P* value
Ischemic time (mean, min)	260.1 ± 52.4	234.4 ± 27.5	.129
Side of allograft			
Right	6 (50.0%)	7 (36.8%)	.710
Left	6 (50.0%)	12 (63.2%)	
Cardiopulmonary bypass	5 (41.7%)	1 (5.3%)	.022
ECMO requirement	2 (16.7%)	0	.142
Chest left open after transplant	3 (25%)	0	.049
Early reoperation (bleeding/tracheostomy)	6 (50%)	4 (21.1%)	.127

**Table 2:** Followup and outcomes data.

Variable	Aborted bilateral (*n* = 12)	Planned single (*n* = 19)	*P* value
Primary graft dysfunction (grade 3)	6 (50.0%)	4 (21.1%)	.127
Length of mechanical ventilation (days)	2 (IQR: 1–41)	2 (IQR: 1-2)	.120
Length of stay in ICU (days)	4 (IQR: 2–30)	3 (IQR: 2-3)	.093
Length of stay in hospital (days)	23 (IQR: 9–52)	13 (IQR: 10–19)	.067
Mean followup time (years)	2.0 ± 3.0	3.6 ± 2.4	.38
Intervention for Airway Complication	1 (8.3%)	3 (15.8%)	1.000
KM Survival			
1 year	61.9% (*n* = 6)	84.2% (*n* = 16)	.941
2 year	51.6% (*n* = 5)	79% (*n* = 13)	
3 years	51.6% (*n* = 4)	72.4% (*n* = 10)	

IQR: Interquartile range, KM: Kaplan Meier.
